# Artificial Neural Networks for Predicting the Diameter of Electrospun Nanofibers Synthesized from Solutions/Emulsions of Biopolymers and Oils

**DOI:** 10.3390/ma16165720

**Published:** 2023-08-21

**Authors:** Guadalupe Cuahuizo-Huitzil, Octavio Olivares-Xometl, María Eugenia Castro, Paulina Arellanes-Lozada, Francisco J. Meléndez-Bustamante, Ivo Humberto Pineda Torres, Claudia Santacruz-Vázquez, Verónica Santacruz-Vázquez

**Affiliations:** 1 Facultad de Ingeniería Química, Benemérita Universidad Autónoma de Puebla, Av. San Claudio y 18 Sur, Puebla 72570, Mexico; guadalupe.cuahuizo@alumno.buap.mx (G.C.-H.); octavio.olivares@correo.buap.mx (O.O.-X.); paulina.arellanes@correo.buap.mx (P.A.-L.); 2 Centro de Química, Instituto de Ciencias, Benemérita Universidad Autónoma de Puebla, Av. San Claudio y 18 Sur, Puebla 72570, Mexico; mareug.castro@correo.buap.mx; 3 Laboratoria de Química Teórica, Centro de Investigación, Deptartamento de Fisicoquímica, Facultad de Ciencias Químicas, Benemérita Universidad Autónoma, Av. San Claudio y 18 Sur, Puebla 72570, Mexico; francisco.melendez@correo.buap.mx; 4 Facultad de Ciencias de la Computación, Benemérita Universidad Autónoma de Puebla, Av. San Claudio y 14 Sur, Puebla 72570, Mexico; ivopinedatorres@gmail.com

**Keywords:** biopolymers, essential oil, network configuration, Levenberg–Marquardt backpropagation, SEM

## Abstract

In the present work, different configurations of nt iartificial neural networks (ANNs) were analyzed in order to predict the experimental diameter of nanofibers produced by means of the electrospinning process and employing polyvinyl alcohol (PVA), PVA/chitosan (CS) and PVA/*aloe vera* (Av) solutions. In addition, gelatin type A (GT)/alpha-tocopherol (α-TOC), PVA/olive oil (OO), PVA/orange essential oil (OEO), and PVA/anise oil (AO) emulsions were used. The experimental diameters of the nanofibers electrospun from the different tested systems were obtained using scanning electron microscopy (SEM) and ranged from 93.52 nm to 352.1 nm. Of the three studied ANNs, the one that displayed the best prediction results was the one with three hidden layers with the flow rate, voltage, viscosity, and conductivity variables. The calculation error between the experimental and calculated diameters was 3.79%. Additionally, the correlation coefficient (R^2^) was identified as a function of the ANN configuration, obtaining values of 0.96, 0.98, and 0.98 for one, two, and three hidden layer(s), respectively. It was found that an ANN configuration having more than three hidden layers did not improve the prediction of the experimental diameter of synthesized nanofibers.

## 1. Introduction

Currently, the application of electrospun veils covers a wide range of uses in different industries such as pharmaceutical, food, and environmental due to the special physicochemical and microstructural characteristics displayed by these materials [1]. In the last years, different methods such as melt spinning, dissolution, emulsion, and electrospinning have been proposed for producing micro and nanopolymeric fibers. However, the latter is the most employed technique because its versatility enables the production of veils consisting of continuous fibers with tailorable porous structures [2,3,4]. Due to the growing interest in the electrospinning field, the fibers achieved using this method are being applied in areas such as nanocatalysis, filtration, biotechnology, and environmental engineering, among others [5,6,7,8]. It has been reported that characteristics such as diameter, morphology, porosity, alignment, and mechanical properties of electrospun fibers vary as functions of the electrospinning conditions such as the solution applied voltage, volumetric flow, and injector–collector distance. It is also known that the physical properties of the polymeric solution such as viscosity, conductivity, surface tension, and concentration in addition to ambient parameters such as temperature, atmospheric pressure, and relative humidity are important electrospinning factors [9,10,11]. The application field of the electrospinning process is vast and complex, where surface phenomena such as electric current (electrostatic) [12,13], momentum, and mass [14] transfer take place. The advances in the ANN field along with the use of mathematical models have allowed descriptive and predictive modeling of the physical characteristics of electrospun fibers [15]. Some authors have established the use of different mathematical models such as response surface, linearization of equations from experimental data, and the development of empirical equations for predicting the diameter behavior as a function of process variables [16,17]; notwithstanding, the error percentage is significant, because experimental data are employed for producing biopolymer nanofibers with different viscosity, conductivity, flow, voltage, and diameter features. Since this process implies a wide database, the development and use of an ANN is a viable option for predicting the experimental diameter of nanofibers. In this sense, Nasouri [18] proposed a model for predicting the diameter of nanofibers from PVP solutions (polyvinylpyrrolidone) by employing an artificial intelligence system. The proposed model consists of three neurons in the input layer (PVP concentration, applied voltage, and needle-to-collector distance), five neurons in hidden layers with a tangent sigmoid transfer function, and one neuron in the output layer (average diameter of PVP nanofibers) with a linear transfer function. The hidden layer is an intermediate layer between the input and output layers of the network, which is composed of neurons that transfer information to other neurons and improve the prediction of the output parameter.

This study reported a correlation coefficient (R^2^) of 0.98, concluding that the parameters used in the design of the employed model significantly affected the average diameter of the electrospun nanofibers. Maurya et al. [19] established the importance of the relationship between the parameters of the electrospinning process and the diameter of magnetic nanofibers of ferrofluid/polyvinyl alcohol (PVA) using artificial neural networks (ANNs). The ANN model was trained with a backpropagation algorithm and sigmoid activation function in the hidden layers and a learning rate of 0.6. The optimal configuration was obtained by testing one and two hidden layers in the first case, where the hidden neurons were varied from 2 to 15. The layer with thirteen hidden neurons achieved a minimum mean square error (MMSE) of 8.9 × 10^−5^. By employing two hidden layers, the minimal MMSE value was 8.5 × 10^−5^, which was obtained by using eight hidden neurons. Since the error values were minimal with two hidden layers, the configuration 4-8-8-1 was selected for maximal optimization, reporting R^2^ values of 0.79 and 0.94 for the training and testing data, respectively. Lakshmi Narayana et al. [20] developed an ANN in order to predict and analyze the diameter of polycaprolactone (PCL) fibers as a function of the parameters of the 3D melt electrospinning process. The model employed the backpropagation algorithm for training and process variables such as collector rate, tip-to-nozzle distance, applied pressure, voltage, and average microfiber diameter (output variable) were considered. The best-reported ANN consisted of a hidden layer with three neurons and a sigmoid activation function in the hidden layers with a learning rate of 0.3. By putting into practice this model, an R^2^ value of 0.97 was obtained for the training data and 0.98 for the set of test data. Likewise, Premasudha et al. [21] presented a more complex model for predicting the diameter of polysaccharide (Hylon VII starch)-based biopolymer nanofibers as a function of the polymer concentration, solution feed flow, applied voltage, injector–collector distance, two-output-layer neurons, nanofiber diameter, and good, regular, and bad quality classification. The ANN model was trained with a backpropagation algorithm, using a sigmoid activation function in the hidden layers and a learning rate of 0.4. The optimal reported configuration consisted of two hidden layers with eight neurons in each layer (4-8-8-2). The results indicated classification and prediction accuracy of 93.9 and 95.2%, respectively. It is known that the study of polymeric solutions is widely spread [22,23,24,25]; however, the application of nanofibers produced from polymeric emulsions has been slightly researched, where just a few authors have reported the use of vegetable essential oils [26,27,28,29]. In addition, ANN models for predicting the diameter of nanofibers from polymeric emulsions with essential oils (EOs) are scarce. The importance of considering EO stems from features such as their biocide activity, application as food preservatives and additives, and antioxidant and antimicrobial properties, among others [30,31]. In the present work, the construction of an ANN model for understanding the nonlinear relationship among electrospinning operation variables (flow rate, voltage, and solution/emulsion properties) for systems of biopolymer solutions/emulsions (PVA, CS, GT, and EO) is studied in order to predict the average experimental diameter of electrospun nanofibers, thus reducing the experimental stages.

## 2. Materials and Methods

### 2.1. Preparation of the Electrospinning Solutions/Emulsions

Table 1 shows the preparation procedure of different aqueous polymeric solutions/emulsions whose physical properties were established before the electrospinning process. The following conditions of the electrospinning process were considered: composition, voltage, flow rate, viscosity, conductivity, and experimental diameter of the nanofibers.

The PVA solutions were prepared by dispersing them in distilled water with stirring at 600 rpm and 80 ± 1 °C for 30 min [32,33]. Afterward, the EO (OO, OEO, or AO) was added at 25 ± 1 °C to form the emulsion, which was mechanically stirred for 5 min.

The GT solution was dispersed in acetic acid and distilled water solution and stirred at 600 rpm and 90 ± 1 °C for 1 h. Then, α-TOC was incorporated at 25 ± 1 °C and emulsified by mechanical stirring for 5 min [34]. As for the PVA/CS solutions, a solution at 10% *w*/*w* and another of CS employing an aqueous solution of acetic acid at 2% were prepared; then, both solutions were mixed up at 25 ± 1 °C for 1 h prior to the electrospinning process [35]. Finally, the PVA/Av solutions were homogenized at 25 ± 1 °C for 1 h.

The solutions, at room temperature, were injected into a SEV electrospinning piece of equipment (model espin-50 kV). For the electrospinning process, a voltage interval ranging from 20 to 30 kV was selected; the apparatus was connected in series, and the injector–collector distance oscillated between 10 and 20 cm.

### 2.2. Characterization of the Solutions/Emulsions

The viscosity of the studied solutions/emulsions was determined by means of a RheolabQC Anton Paar rheometer at 25 ± 1 °C, employing the DG24 concentric cylinder configuration and the Star Rheoplus 3.0x software. The conductivity was measured with a Conductronic piece of equipment (model PC18) at 25 ± 1 °C.

The viscosity and conductivity of the PVA emulsions incorporated with the EOs and GT/α-TOC emulsions and PVA/CS and PVA/Av solutions were established prior to the electrospinning process.

### 2.3. Morphological Study of the Electrospun Nanofibers

The structures of the electrospun nanofibers were analyzed by means of scanning electron microscopy (SEM), employing a microscope (model JSM-6610LV). The images were processed with the ImageJ 1.51j8 software, and with it, the experimental diameter of the nanofibers was also determined, which corresponded to the arithmetic mean of 85 measurements of SEM carried out with different nanofibers [36].

### 2.4. Structure of the ANN Model

In the present work, the multilayer perceptron (MLP) neural network was employed with sigmoid activation (logsing) in the hidden layers, according to Equation (1), and linear in the output layer, as indicated in Equation (2) [37,38]:

(1) $$f\left(x\right)=\frac{1}{1+e^{-x}}\;$$

(2) g(x)=x  

The experimental data that were fed into the ANN were normalized by means of Equation (3) [39]:

(3) $$x_{n}=\frac{x-mean\left(x\right)}{sd\left(x\right)}\;\;\;$$

where $x_{n}$ is the normalized value of x input variables, mean (x) is the mean of x, and sd(x) is the standard deviation of x. The training of the ANN was carried out by means of the Levenberg–Marquardt backpropagation (trainlm) algorithm with a learning rate of 0.4. The experimental data were divided into three groups: training, validation, and test sets with around 70%, 15%, and 15% of the total set of experimental data, respectively. The computations were performed with the Matlab software version 2018b. Four neurons corresponding to four electrospinning variables (flow rate, voltage, viscosity, and conductivity) were employed in the input layer and one neuron ascribed to the diameter of the electrospun nanofiber was set in the output layer. Different configurations were tested for one, two, and three hidden layers, as shown in Table 2. The configuration of the ANN model was selected based on the minimal MMSE value of the training, test, and validation sets [40,41].

The error percentage was calculated by means of Equation (4):

(4) $$\%error=\left|\frac{D_{predicted}-D_{experimental}}{D_{experimental}}\right|\times 100$$

### 2.5. Computation of the Relative Contribution of the Input Variables

The relative contribution of each input variable was calculated by means of the algorithm proposed by Olden et al. [42], according to Equation (5):

(5) $$CR_{P}=\frac{\sum_{j=1}^{n}\frac{\left|w_{jp}||v_{j}\right|}{\sum_{k=1}^{I}|w_{jk}|}}{\sum_{i=1}^{I}\sum_{j=1}^{n}\frac{\left|w_{jp}||v_{j}\right|}{\sum_{k=1}^{I}|w_{jk}|}}\;$$

where $CR_{P}$ is the contribution percentage by each input at the ANN output, p is the variable input for knowing its relative contribution, n is the number of hidden neurons, j is the j-th hidden neuron, I is the number of ANN inputs, $w_{jp}$ is the synaptic weight of the p input toward neuron j, $v_{j}$ is the synaptic weight of neuron j toward the output, and $w_{jk}$ is the synaptic weight of the input k toward neuron j.

## 3. Results and Discussion

### 3.1. Morphologic Characterization

The experimental diameters of the nanofibers, reported in the support material (Table S1), are close to those obtained by [43] for PVA electrospun nanofibers at 8 and 10%, whose diameters measured 270 and 390 nm, respectively.

**Figure 1:**
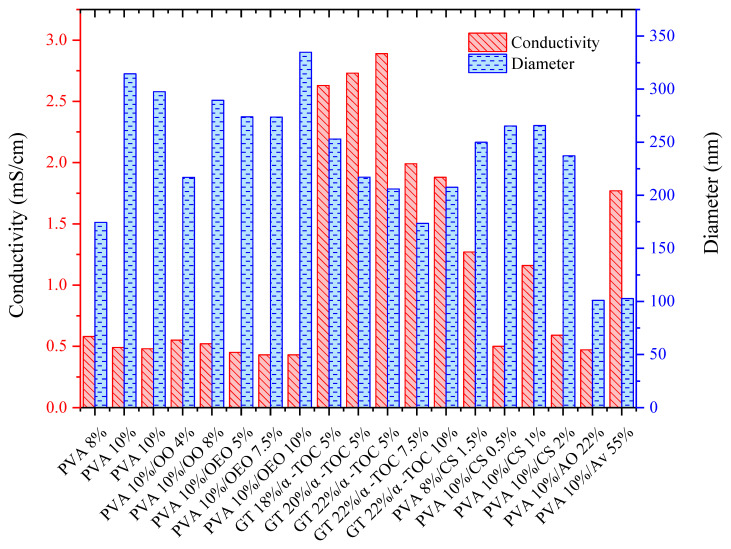
Conductivity and fiber diameter vs. composition of solutions/emulsions.

As for the PVA/OEO nanofibers, a directly proportional relationship between the concentration and experimental diameter of the nanofibers was observed, as shown in Figure 1. This effect was similar to the one on the PVA/OO nanofibers. Notwithstanding, for the PVA/OEO system, an inverse behavior was identified between the conductivity and nanofiber experimental diameter because the OEO encapsulated in the nanofibers, which contains d-limonene, monoterpenes, trans-dihydrocarvone, and trans-p-methane, among other hydrophobic compounds, diminished the sample conductivity [33,44].

On the other hand, a higher concentration of gelatin in the solution of the GT/α-TOC nanofibers allowed the production of thinner nanofibers, as observed in Figure 1; this result can be attributed to the use of acetic acid during the preparation of the protein solution prior to the electrospinning process. It should be kept in mind that gelatin is an amphoteric protein that consists of amino acids such as glycine, alanine, proline, and hydroxyproline, in addition to other residual monomers that are positively charged, derived from $NH_{3}^{+}$ and carboxyl radicals, which promote high conductivity in the emulsion, which depends on the solution pH and concentration of solvents such as acetic acid [45,46,47].

Regarding the PVA/CS nanofibers, a decrease in the experimental diameter of the nanofibers was observed as the CS concentration increased, as shown in Figure 1. Furthermore, the conductivity was increased, which has been reported by several authors [48,49]; for example, Chen et al. [50] studied electrospun solutions with different conductivities and investigated the effect on the morphology of the nanofibers, finding that the higher the conductivity, the smaller the nanofiber diameter.

On the other hand, the Av extract consists of aloine (polyhydroxylated anthraquinone glucoside), which is characterized by having a long alkyl structure that explains the high electrical conductivity of the electrospun solution [51].

As observed in Table S1, the values of the input variables are expressed in different units and magnitudes; for this reason, it is necessary to normalize the data in order for the variables to be similar even when they belong to different distributions. Normalization, prior to the ANN training, is important for producing satisfactory results and reducing the computation time [23,52].

**Figure 2:**
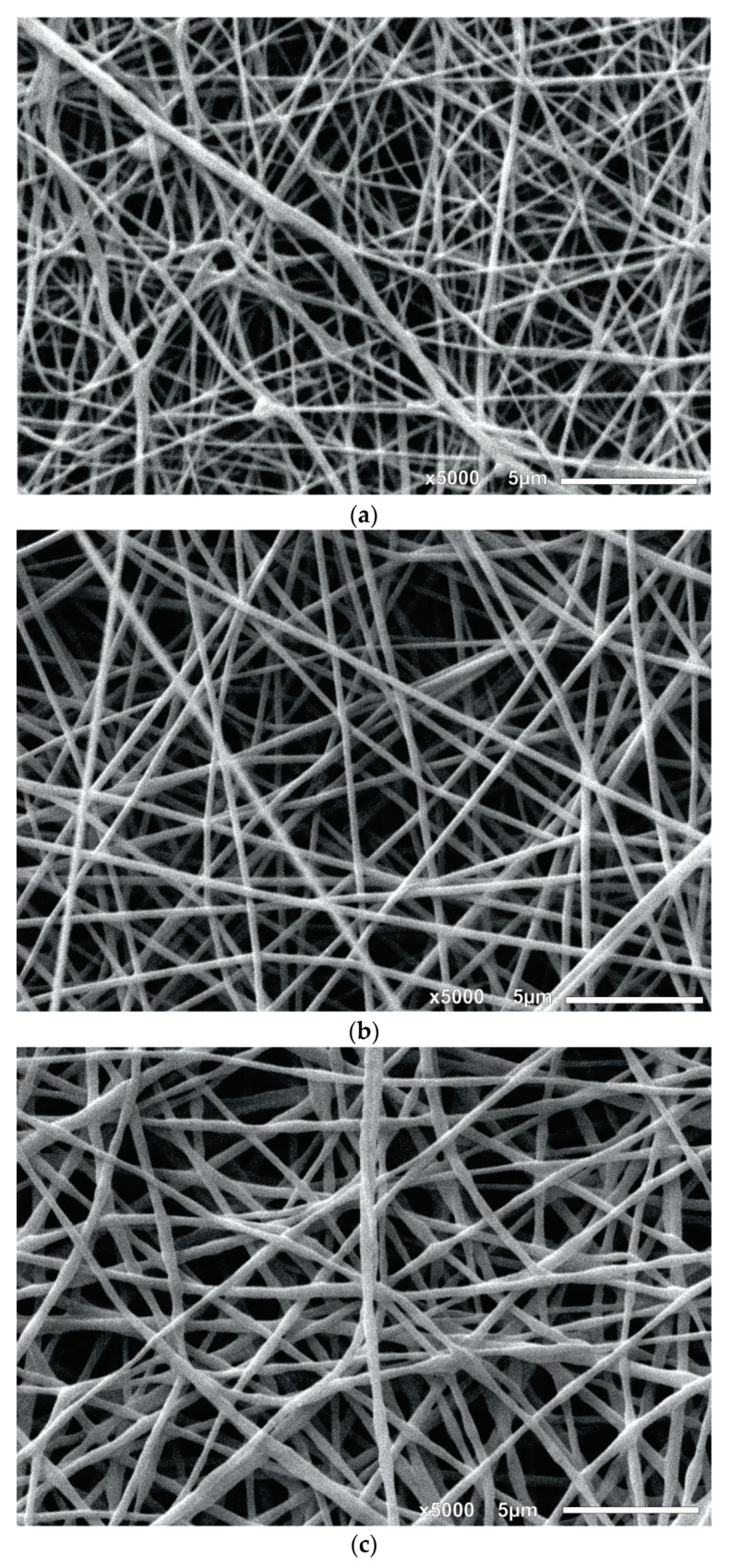

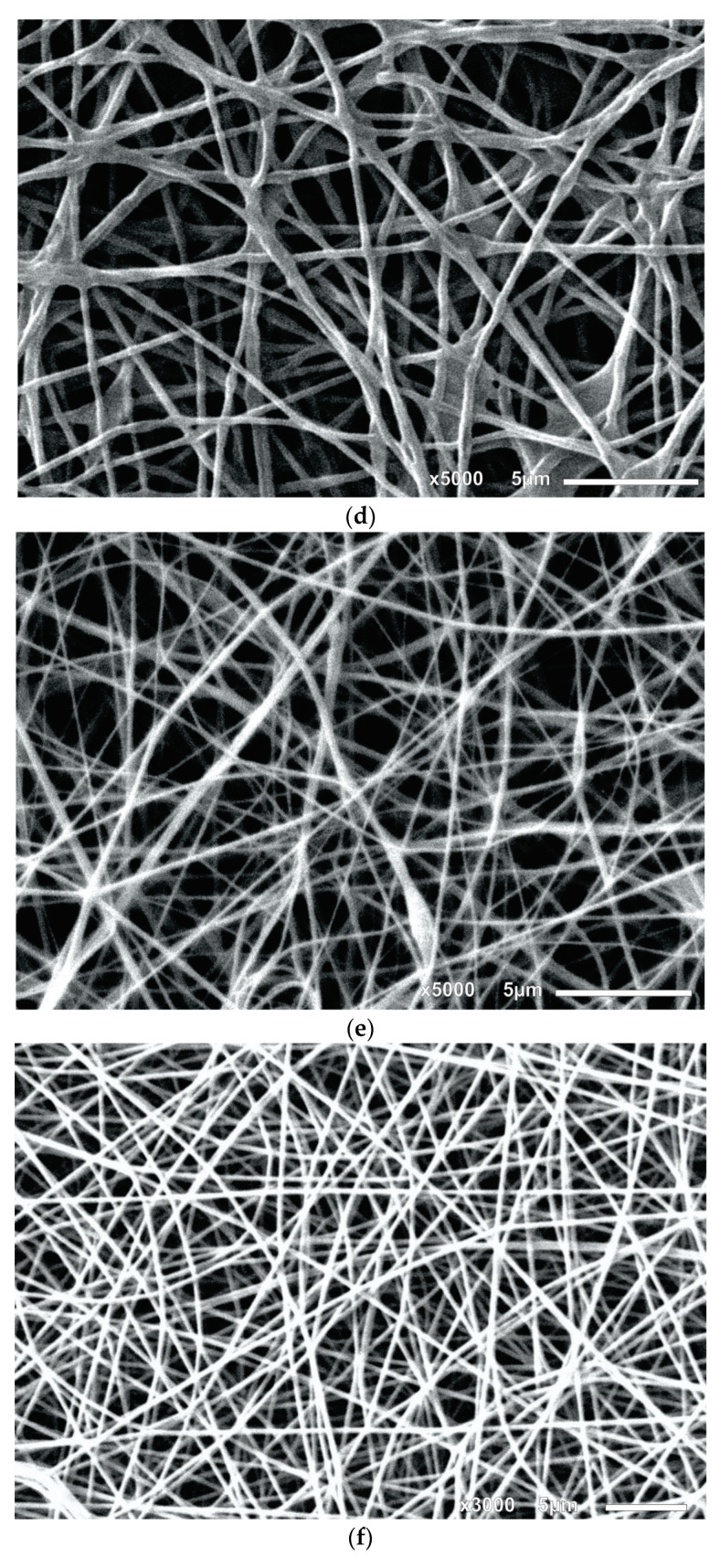

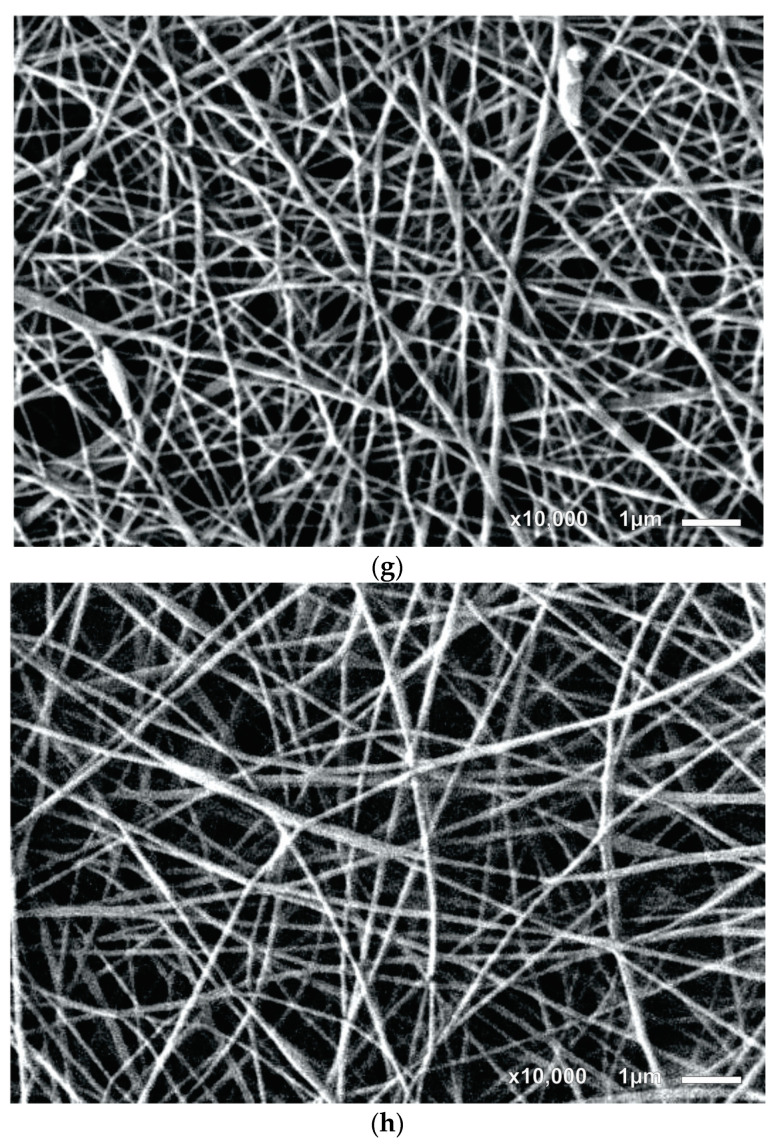
Micrographs of the studied systems: (**a**) 8% PVA, (**b**) 10% PVA, (**c**) PVA/OO, (**d**) PVA/OEO, (**e**) GT/α-TOC, (**f**) PVA/CS, (**g**) PVA/AO, and (**h**) PVA/Av.

Figure 2 shows the SEM micrographs of some of the studied systems produced by the electrospinning process. In Figure S1, micrographs with different magnifications are shown. Figure 2a,b correspond to the solution prepared with PVA (8 and 10% *w*/*w*), where it is evidenced that the PVA concentration plays a major role in the surface topography and distribution of the nanofiber experimental diameter. It is confirmed that at 10% PVA, larger diameters than those with 8% are produced; also, the presence of a branched microstructure is evidenced. The micrographs in Figure 2c–h correspond to the electrospun nanofibers from the solutions/emulsions, where it can be observed that the nanofibers display both homogeneous and uniform topography and diameter distribution. These results indicate that the experimental conditions of the electrospinning process allowed the production of well-defined nanofiber shapes, which will be predicted by means of the proposed ANN configurations.

### 3.2. Architectures with One Hidden Layer

The weighing of the input variables (Table 3) and output variable, which was the average nanofiber diameter of each sample, was optimized in order to identify the best structure as a function of the number of layers and neurons. Due to the sensitivity of the prediction computation of the experimental diameter of the nanofibers, the optimal ANN structure was identified as a function of the number of hidden layers and neurons; this procedure has also been reported by other authors [53,54]. The optimal ANN configuration was selected according to the MMSE and R^2^ that relates the predicted diameter to the experimental one. For test 1, the R^2^ value was 0.71, and for test 2, it was 0.70, which indicated that the results of tests 1 and 2 were not satisfactory in the prediction of the experimental nanofiber diameter; for this reason, increasing the number of input variables to four was considered, as shown in Table 3.

**Figure 3:**
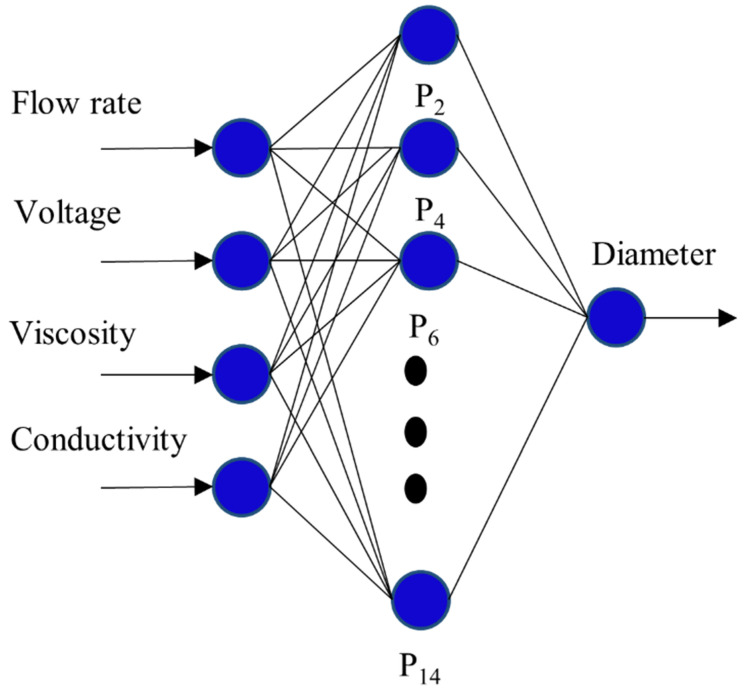
Configuration of a single tested hidden layer.

The configuration with four ANN input variables consisted of one hidden layer with a variable number of neurons from two to fourteen, as shown in Figure 3. The obtained results are shown in Table 4, which revealed two important possibilities in the prediction of the experimental nanofiber diameter as a function of the number of neurons in the hidden layer. Firstly, if the number is too small, the model cannot yield an accurate output value. Secondly, it was observed that an increase in the number of neurons in the hidden layer did not guarantee a better estimation of the output experimental data; it can even produce overfitting, which prevents the generalization process during the test phase, thus generating an overparameterized model. These problems in the use of ANN to predict experimental data have also been reported by other authors [55].

In order to avoid overparameterization, a variable number of neurons in a hidden layer from 2 to 14 was chosen for this study. Higher test MMSE values were observed in the ANN configurations consisting of 2, 4, 12, and 14 neurons, as observed in Table 4. On the other hand, it is shown that the 8-neuron configuration with training, test, and validation displayed lower MMSE values (0.03, 0.08, and 0.04, respectively) with an R^2^ of 0.96 and an error percentage of 4.44%; these results are in contrast with the rest of the proposed configurations for predicting the experimental nanofiber diameter, thus confirming that this configuration was the best approach to the experimental values of the nanofiber diameters obtained by SEM. Different configurations of ANN models featuring a single hidden layer have been employed in the prediction of experimental data [39,56], which is the case of the work by You et al. [57], who employed this configuration type to predict the molecular weight of polycaprolactone synthesized through enzymatic polymerization; more specifically, a configuration featuring one hidden layer with 20 neurons was used, obtaining an R^2^ of 0.99.

### 3.3. Sensitivity Analysis

It has been reported that the use of a single hidden layer can favor the prediction of experimental data [58]; however, the ANN accuracy can be affected and, as a consequence, its training can be deficient, i.e., if a bad fitting process takes place with R^2^ ≤ 0.7, more training with the concomitant modification of both hidden layers and neurons is necessary [59,60].

**Figure 4:**
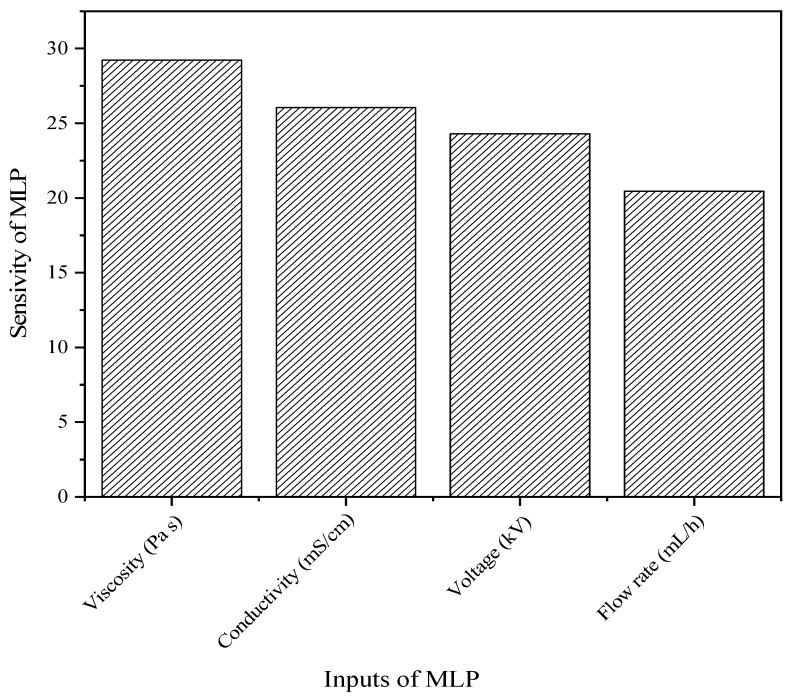
Importance of each input variable in the experimental nanofiber diameter.

**Figure 5:**
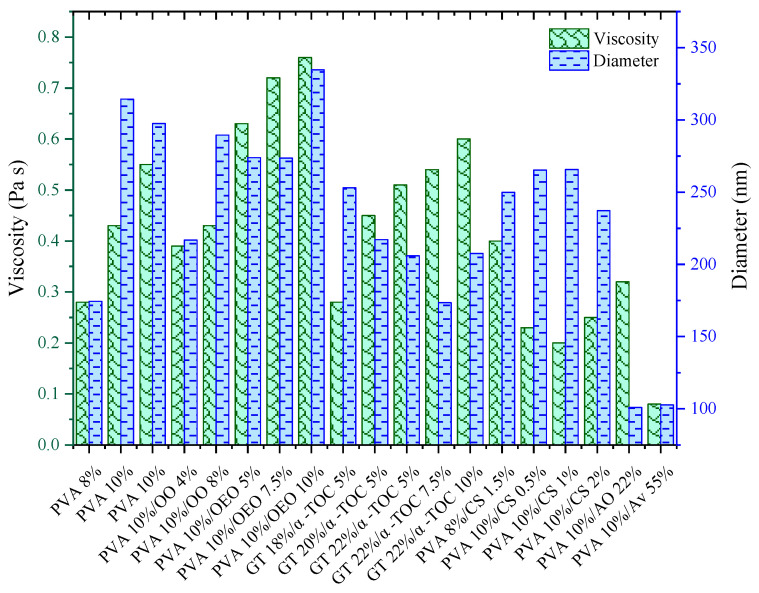
Viscosity and fiber diameter vs. composition of solutions/emulsions.

For this reason, the 8-neuron configuration allowed the establishment of the importance of the variables related to the characteristics of the polymeric solutions/emulsions and of those operation variables associated with the electrospinning process that are implied in the prediction of the experimental diameter of nanofibers. This type of sensitivity analysis was also proposed by Nasouri [18] and Kalantary et al. [16], where the experimental input variables (concentration and distance) were more important in the diameter prediction of PVP (polyvinylpyrrolidone) and poly(3-caprolactone)/gelatin nanofibers; in order to be able to identify the most important variables, the authors fed the synaptic weight of the ANN inputs and outputs of the optimized ANN by means of Equation (5). Figure 4 shows the relative contribution of the one-hidden-layer configuration. It can be observed that the viscosity of the solutions/emulsions is the most important input variable in the diameter prediction with 29%, i.e., the higher the viscosity, the larger the experimental nanofiber diameter, as observed in Figure 5. These results are in good agreement with the experimental data, where the main contribution during electrospinning was given by viscosity. In addition, this variable is related to temperature, composition, and chemical nature of the components of the polymeric solutions/emulsions, whereas the variables with less relevance were evidenced by the conductivity (26%), voltage (24%), and feeding flow rate (20%) data. Furthermore, it was found that the increase in the EO concentration augmented the viscosity and decreased the conductivity of the emulsions, as observed in Table S1; similar results were reported by Kalantary et al. [16], confirming that the highest weight in the prediction of the nanofiber diameters corresponded to the polymer concentration in the electrospinning solution. On the other hand, Keirouz et al. [61] established a directly proportional relationship between the nanofiber diameter and viscosity. Likewise, Ibrahim et al. [62] proposed an inversely proportional relationship between conductivity and diameter.

### 3.4. Architecture with Two Hidden Layers

Once the response of one hidden layer predicting the diameter of nanofibers was analyzed, the study of the effect of two hidden layers was proposed in order to obtain an R^2^ closer to unity, which would imply an enhanced relationship between the experimental diameters and those predicted by the ANN. To this end, the 8-*n* configuration was proposed, which consisted of 8 neurons in the first hidden layer, whereas *n* (4, 8, 12, 16, and 20) represented the variations of the neurons corresponding to the second hidden layer, with the following configurations shown in Figure S2: 8-4, 8-8, 8-12, 8-16 and 8-20. From the analysis of the MMSE and R^2^ of the output results corresponding to the training, test, and validation stages, it was observed that the configurations in Figure S2c (8–12) and Figure S2d (8-16) presented similar results, which were the lowest of all the configurations, as shown in Table 5. The following MMSE values for each stage for Figure S2c (0.02, 0.05, 0.04) and Figure S2d (0.02, 0.06, 0.03) were obtained, which revealed MMSE values in the configuration in Figure S2c that were similar to those in (d); however, the configuration (8-16) in Figure S1d showed more efficiency in the prediction of the experimental nanofiber diameter with an R^2^ of 0.98 and average error percentage of 3.96%. Similar results were presented by Khatti [52], who employed a configuration consisting of two hidden layers with eleven and five neurons to predict the experimental diameter of polycaprolactone nanofibers with an R^2^ of 0.97. The prediction of the experimental nanofiber diameter by means of this ANN is complex and depends on the studied system and availability and quality of the input data.

### 3.5. Architecture with Three Hidden Layers

**Figure 6:**
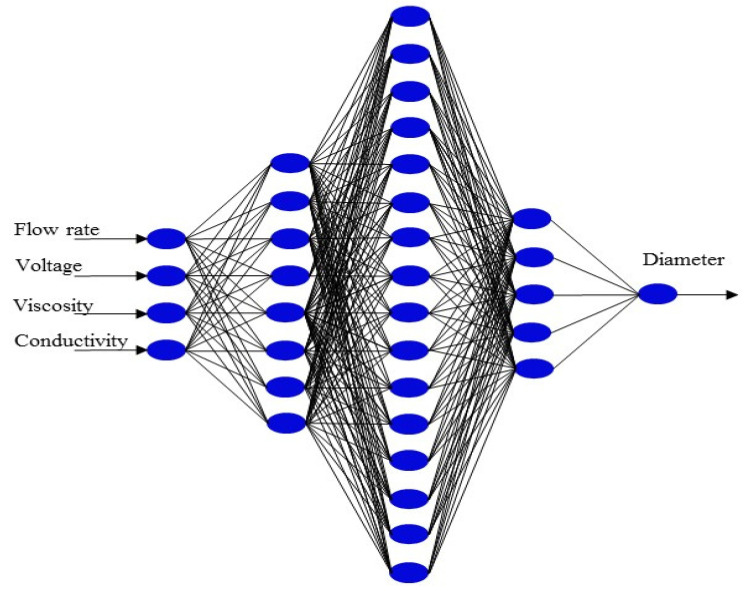
Configuration of the ANN with the best fitting between the experimental and predicted data.

The aim of analyzing the configuration with two hidden layers (8-16) was to increase the R^2^ through an additional configuration of three hidden layers of 8-16-*n* neurons, as shown in Figure S3, where *n* varied from three to six. The results revealed that the 8-16-3 (Figure S3a) and 8-16-5 configurations (Figure 6) presented R^2^ values of 0.98 and 0.98, respectively, as shown in Table 6.

As for the test MMSE values, the 8-16-5 configuration presented values that were lower than those of the other configurations. This result means that the prediction of the experimental diameters of the nanofibers by means of one, two, and three hidden layers is reliable according to an R^2^ of 0.98 (Table 7), as reported elsewhere [63]. Furthermore, a significant difference in the prediction of diameters between two and three hidden layers, as shown in Table 7, was not observed. Similar results have been found by employing two and three hidden ANN layers [64,65].

**Figure 7:**
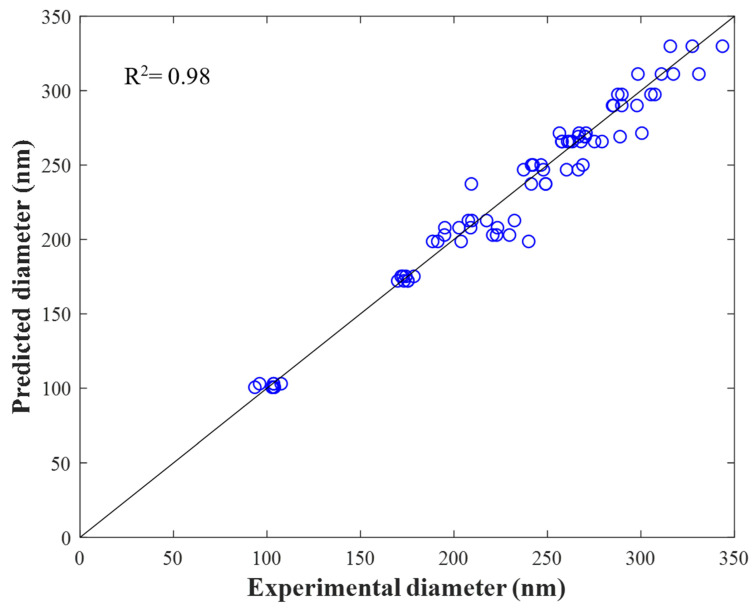
Experimental nanofiber diameters using the electrospinning process vs nanofiber diameters predicted by the ANN with configuration 8-16-5.

Figure 7 shows the distribution of the nanofiber diameters predicted with the 8-16-5 configuration, according to Figure 6, which confirms that the employed ANN is reliable for predicting the experimental diameter obtained by means of the electrospinning of both the PVA, PVA/CS, and PVA/Av solutions and GT/α-TOC, PVA/OO, PVA/OEO, and PVA/AO emulsions. From these results, it can be said that the ANN model with two and three hidden layers contributes to understanding the hierarchy that experimental variables have in the prediction of the experimental diameter, which can help shorten the experimentation times.

## 4. Conclusions

In this work, different configurations of artificial neural networks (ANNs) were used for predicting the experimental diameter produced during the electrospinning process of nanofibers from PVA, PVA/CS, and PVA/Av solutions and GT/α-TOC, PVA/OO, PVA/OEO, and PVA/AO emulsions. In addition, the experimentation is related to the theory by means of an ANN model designed for understanding the nonlinear relationship among operative variables of the electrospinning process such as flow and voltage and physical properties of the biopolymer and essential oil solutions/emulsions.

An enhanced prediction of the experimental nanofiber diameter was achieved by employing four input variables (flow, voltage, viscosity, and conductivity) in comparison with the use of three variables (flow rate, voltage and viscosity or conductivity, voltage, and viscosity) in the ANN configuration.

The ANN configurations that best predicted the experimental nanofiber diameter were those with two and three hidden layers, with an R^2^ close to 0.98. Nevertheless, according to the MMSE, the best configuration for predicting the experimental nanofiber diameter with the test data was the one with three hidden layers, with an MMSE value of 0.03, which is in contrast with the MMSE value of 0.06 for two hidden layers (8-16).

The computation error between the experimental nanofiber diameter obtained by SEM and the one predicted by the ANN was 3.79% for the configuration with three hidden layers, whereas for the one with two layers, it was 3.95%, which confirms the reliability of the ANN with three hidden layers, which will allow for narrowing down the combinations of polymers, solvents, and concentrations of solutions/emulsions in earlier stages in order to optimize the experimentation stage before proceeding to the electrospinning process.

Additionally, it was confirmed that the most influential variable in the prediction of the experimental nanofiber diameter by employing a one-hidden-layer configuration with eight neurons was the viscosity of the solutions/emulsions to be electrospun.

## Figures and Tables

**Table 1:** Methodologies for preparing the electrospinning solutions/emulsions.

Solutions/Emulsions	Procedure
PVA	Prepared PVA aqueous solution (% *w*/*w*): 8 and 10.
PVA/OO	Emulsion formed with 10 (% *w*/*w*) of PVA with OO. Composition (% *w*/*w*): 96 (PVA) with 4 (OO) and 92 (PVA) with 8 (OO).
PVA/OEO	Emulsion formed with 10 (% *w*/*w*) of PVA with OEO. Composition (% *w*/*w*): 95 (PVA) with (OEO), 92.5 (PVA) with 7.5 (OEO), and 90 (PVA) with 10 (OEO).
GT/α-TOC	Emulsions of GT in acetic acid (AA) and distilled water (W). Compositions (% *w*/*w*): [18 g GT; 5 α-TOC; 30 AA; 47 W]; [20 g GT; 5 α-TOC; 30 AA; 45 W]; [22 g GT; 5 α-TOC; 30 AA; 43 W]; [22 g GT; 7.5 α-TOC; 30 AA; 41 W]; [22 g GT; 10 α-TOC; 30 AA; 38 W].
PVA/CS	Solutions with composition (% *w*/*w*): 10 (PVA) with 0.5 (CS), 10 (PVA) with 2 (CS), 10 (PVA) with 1 (CS), and 8 (PVA) with 1.5 (CS).
PVA/AO	Emulsions with composition (% *w*/*w*): 10 (PVA) with 22 (AO).
PVA/Av	Solutions with composition (% *w*/*w*): 10 (PVA), 90 (A) and 55 (Av).

**Table 2:** Tested configurations employing one, two, and three hidden layers in the ANN.

Input Variables	Number of Neurons		
	Layer 1	Layer 2	Layer 3
Flow rate Voltage Viscosity Conductivity	2 4 6 8 10 12 14	4 8 12 16 20	3 4 5 6

**Table 3:** ANN input variables for the prediction of the diameter of the electrospun nanofibers.

Test	Number of Variables	Variables
1	3	Flow rate, voltage, and viscosity
2	3	Conductivity, voltage, and viscosity
3	4	Flow rate, voltage, viscosity, and conductivity

**Table 4:** Configurations tested by employing one ANN hidden layer with input variables such as flow rate, voltage, viscosity, and conductivity.

Number of Neurons		2	4	6	8	10	12	14
R^2^	Training	0.88	0.96	0.97	0.97	0.97	0.97	0.98
	Test	0.63	0.43	0.66	0.82	0.80	0.59	0.64
	Validation	0.70	0.95	0.93	0.96	0.97	0.96	0.96
	Total	0.84	0.92	0.95	0.96	0.95	0.95	0.96
MMSE	Training	0.13	0.04	0.03	0.03	0.03	0.03	0.02
	Test	0.23	0.27	0.12	0.08	0.10	0.15	0.13
	Validation	0.21	0.03	0.04	0.04	0.03	0.03	0.03

**Table 5:** Configurations tested by employing two ANN hidden layers with flow rate, voltage, viscosity, and conductivity as input variables.

Number of Neurons		8-4	8-8	8-12	8-16	8-20
R^2^	Training	0.98	0.97	0.98	0.98	0.98
	Test	0.62	0.82	0.84	0.86	0.88
	Validation	0.96	0.96	0.95	0.98	0.97
	Total	0.95	0.96	0.97	0.98	0.97
MMSE	Training	0.03	0.03	0.02	0.02	0.02
	Test	0.20	0.08	0.05	0.06	0.09
	Validation	0.04	0.04	0.04	0.03	0.03

**Table 6:** Configurations tested by employing three ANN hidden layers with flow rate, voltage, viscosity, and conductivity as input variables.

Number of Neurons		8-16-3	8-16-4	8-16-5	8-16-6
R^2^	Training	0.99	0.98	0.98	0.97
	Test	0.92	0.88	0.93	0.87
	Validation	0.97	0.93	0.96	0.97
	Total	0.98	0.97	0.98	0.96
MMSE	Training	0.02	0.03	0.02	0.03
	Test	0.04	0.05	0.03	0.05
	Validation	0.03	0.03	0.03	0.04

**Table 7:** Selected configurations with four input variables: flow rate, voltage, viscosity, and conductivity.

Number of Hidden Layers		1	2	3
Configurations		8	8-16	8-16-5
R^2^	Training	0.97	0.98	0.98
	Test	0.82	0.86	0.93
	Validation	0.96	0.98	0.96
	Total	0.96	0.98	0.98
MMSE	Training	0.03	0.02	0.02
	Test	0.08	0.06	0.03
	Validation	0.04	0.03	0.03

## Data Availability

The data presented in this study are available on request from the corresponding author.

## Supplementary Materials

The following supporting information can be downloaded at: https://www.mdpi.com/article/10.3390/ma16165720/s1, Table S1: Operation parameters and physical properties of the polymeric solutions/emulsions prior to the electrospinning process and experimental nanofiber diameter; Table S2: Normalized Data; Figure S1: Micrographs of the studied systems: (a) 8% PVA, (b) 10% PVA, (c) PVA/OO, (d) GT/α-TOC and (e) PVA/CS; Figure S2: Configurations with two hidden layers; Figure S3: Configurations with three hidden layers; Figure S4: Diameter distribution of the electrospun nanofibers.
