# Supplementary material for: Artificial Neural Networks for Predicting the Diameter of Electrospun Nanofibers Synthesized from Solutions/Emulsions of Biopolymers and Oils
Source: Materials (Basel). 2023 Aug 21;16(16):5720. doi: 10.3390/ma16165720 (PMC10456520; doi:10.3390/ma16165720)
Supplement: Supplementary file 1 [file materials-16-05720-s001.zip › materials-2543578-supplementary.pdf]

Supplementary Materials

Table S1. Operation parameters and physical properties of the polymeric solutions/emulsions prior to the electrospinning process and experimental nanofiber diameter.

<table>
<tr><th>Solutions/emulsions %(w/w)</th><th>Flow rate (mL/h)</th><th>Voltage (kV)</th><th>Viscosity (Pa·s)</th><th>Conductivity (mS/cm)</th><th>Experimental nanofiber diameter (nm)</th></tr>
<tr><td rowspan="4">PVA 10%</td><td rowspan="16">0.1</td><td rowspan="31">24</td><td rowspan="4">0.43</td><td rowspan="4">0.49</td><td>310.97 ±57.85</td></tr>
<tr><td>298.40 ±42.85</td></tr>
<tr><td>317.34 ±42.61</td></tr>
<tr><td>331.01 ±65.34</td></tr>
<tr><td rowspan="4">PVA 8%</td><td rowspan="4">0.28</td><td rowspan="4">0.58</td><td>178.59 ±22.84</td></tr>
<tr><td>171.75 ±23.77</td></tr>
<tr><td>172.77 ±31.90</td></tr>
<tr><td>174.32 ±30.53</td></tr>
<tr><td rowspan="4">PVA 10%/OO 4%</td><td rowspan="4">0.39</td><td rowspan="4">0.55</td><td>207.69 ±45.28</td></tr>
<tr><td>217.45 ±72.55</td></tr>
<tr><td>209.60 ±57.19</td></tr>
<tr><td>232.37 ±46.93</td></tr>
<tr><td rowspan="4">PVA 10%/OO 8%</td><td rowspan="4">0.43</td><td rowspan="4">0.52</td><td>289.76 ±51.20</td></tr>
<tr><td>285.31 ±29.22</td></tr>
<tr><td>297.87 ±70.49</td></tr>
<tr><td>284.83 ±59.10</td></tr>
<tr><td rowspan="4">PVA 10%</td><td rowspan="4">1.8</td><td rowspan="4">0.55</td><td rowspan="4">0.48</td><td>289.82 ±63.14</td></tr>
<tr><td>307.47 ±69.57</td></tr>
<tr><td>305.36 ±60.22</td></tr>
<tr><td>287.65 ±39.35</td></tr>
<tr><td rowspan="4">PVA 10%/OEO 5%</td><td rowspan="4">0.6</td><td rowspan="4">0.63</td><td rowspan="4">0.45</td><td>270.18 ±56.21</td></tr>
<tr><td>266.49 ±69.46</td></tr>
<tr><td>270.03 ±36.70</td></tr>
<tr><td>288.85 ±44.90</td></tr>
<tr><td rowspan="4">PVA 10%/OEO 7.5%</td><td rowspan="4">1.2</td><td rowspan="4">0.72</td><td rowspan="4">0.43</td><td>300.51 ±55.93</td></tr>
<tr><td>266.92 ±45.22</td></tr>
<tr><td>270.63 ±38.41</td></tr>
<tr><td>256.41 ±34.55</td></tr>
<tr><td rowspan="3">PVA 10%/OEO 10%</td><td rowspan="3">1.8</td><td rowspan="3">0.76</td><td rowspan="3">0.43</td><td>343.61 ±61.47</td></tr>
<tr><td>327.47 ±68.58</td></tr>
<tr><td>352.01 ±51.61</td></tr>
</table>

					315.72 ±67.04
**GT 18%/ α -TOC 5%**	0.02	30	0.28	2.63	247.81 ±51.28
					266.49 ±45.02
					237.26 ±53.50
					260.24 ±59.68
**GT 20%/ α -TOC 5%**			0.45	2.73	194.94 ±34.10
					229.70 ±32.89
					222.82 ±25.30
					220.76 ±30.64
**GT 22%/ α -TOC 5%**			0.51	2.89	239.98 ±46.10
					203.85 ±33.23
					191.33 ±31.29
					188.60 ±30.88
**GT 22%/ α -TOC 7.5%**			0.54	1.99	173.29 ±28.02
					169.90 ±25.86
					175.36 ±22.92
					175.37 ±20.37
**GT 22%/ α -TOC 10%**			0.60	1.88	223.28 ±51.35
					202.68 ±31.80
					208.91 ±44.72
					195.11 ±43.10
**PVA 10%/CS 0.5%**	0.1	26	0.23	0.50	279.18 ±41.62
					257.83 ±60.38
					260.81 ±42.58
					263.34 ±44.75
**PVA 10%/CS 2%**			0.25	0.59	209.32 ±51.89
					249.06 ±56.02
					249.02 ±57.22
					241.35 ±41.19
**PVA 10%/CS 1%**	0.14	21	0.20	1.16	275.18 ±46.51
					257.65 ±51.03
					267.98 ±48.90
					261.79 ±41.89
**PVA 8%/CS 1.5%**	0.1	26	0.40	1.27	268.95 ±36.30
					246.61 ±44.48
					242.36 ±35.06
					241.50 ±26.38
**PVA 10%/AO 22%**	0.6	24	0.32	0.47	103.49 ±19.28
					102.68 ±12.79
					93.52 ±15.87
					103.96 ±17.85

**PVA 10%/Av 55%**	1	24	0.08	1.77	103.51 ±13.74
					103.53 ±14.08
					96.05 ±16.68
					107.58 ±18.15

Table S2. Normalized Data.

Solutions/emulsions %(w/w)	Flow (mL/h)	Voltage (kV)	Viscosity (Pa·s)	Conductivity (mS/cm)	Experimental nanofiber diameter (nm)
**PVA 10%**	-0.54	-0.63	0.04	-0.76	1.20
					1.01
					1.30
					1.52
**PVA 8%**	-0.54	-0.63	-0.80	-0.66	-0.88
					-0.98
					-0.97
					-0.94
**PVA 10%/OO 4%**	-0.54	-0.63	-0.18	-0.69	-0.42
					-0.27
					-0.39
					-0.03
**PVA 10% /OO 8%**	-0.54	-0.63	0.04	-0.73	0.87
					0.80
					1.00
					0.79
**PVA 10%**	2.36	-0.63	0.73	-0.77	0.87
					1.15
					1.11
					0.84
**PVA 10%/OEO 5%**	0.31	-0.63	1.15	-0.81	0.56
					0.50
					0.56
					0.86
**PVA 10%/OEO 7.5%**	1.34	-0.63	1.64	-0.83	1.04
					0.51
					0.57
					0.35
**PVA 10%/OEO 10%**	2.36	-0.63	1.87	-0.83	1.72
					1.46
					1.85

					1.28
**GT 18%/ α -TOC 5%**	-0.68	1.54	-0.80	1.71	0.21
					0.50
					0.05
					0.41
**GT 20%/ α -TOC 5%**	-0.68	1.54	0.13	1.83	-0.62
					-0.07
					-0.18
					-0.21
**GT 22%/ α -TOC 5%**	-0.68	1.54	0.46	2.01	0.09
					-0.48
					-0.68
					-0.72
**GT 22%/ α -TOC 7.5%**	-0.68	1.54	0.65	0.97	-0.96
					-1.01
					-0.93
					-0.93
**GT 22%/ α -TOC 10%**	-0.68	1.54	1.00	0.85	-0.17
					-0.50
					-0.40
					-0.62
**PVA 10%/CS 0.5%**	-0.54	0.10	-1.11	-0.75	0.70
					0.37
					0.42
					0.45
**PVA 10%/CS 2%**	-0.54	0.10	-0.95	-0.64	-0.39
					0.23
					0.23
					0.11
**PVA 10%/CS 1%**	-0.48	-1.71	-1.27	0.02	0.64
					0.37
					0.53
					0.43
**PVA 8%/CS 1.5%**	-0.54	0.10	-0.13	0.14	0.54
					0.19
					0.13
					0.11
**PVA 10%/AO 22%**	0.31	-0.63	-0.57	-0.78	-2.06
					-2.07
					-2.21
					-2.05

**PVA 10%/Av 55%**	1.00	-0.63	-1.91	0.72	-2.06
					-2.06
					-2.17
					-1.99

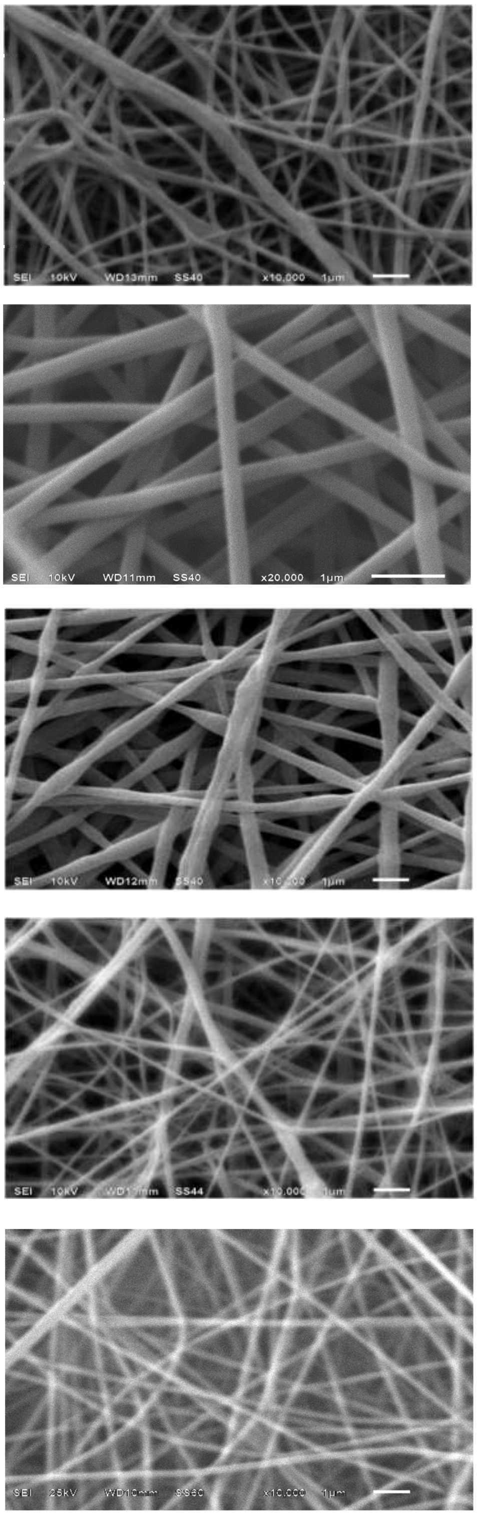

Figure S1. Micrographs of the studied systems: a) 8% PVA, b) 10% PVA, c) PVA/OO, d)GT/ α -TOC and e) PVA/CS.

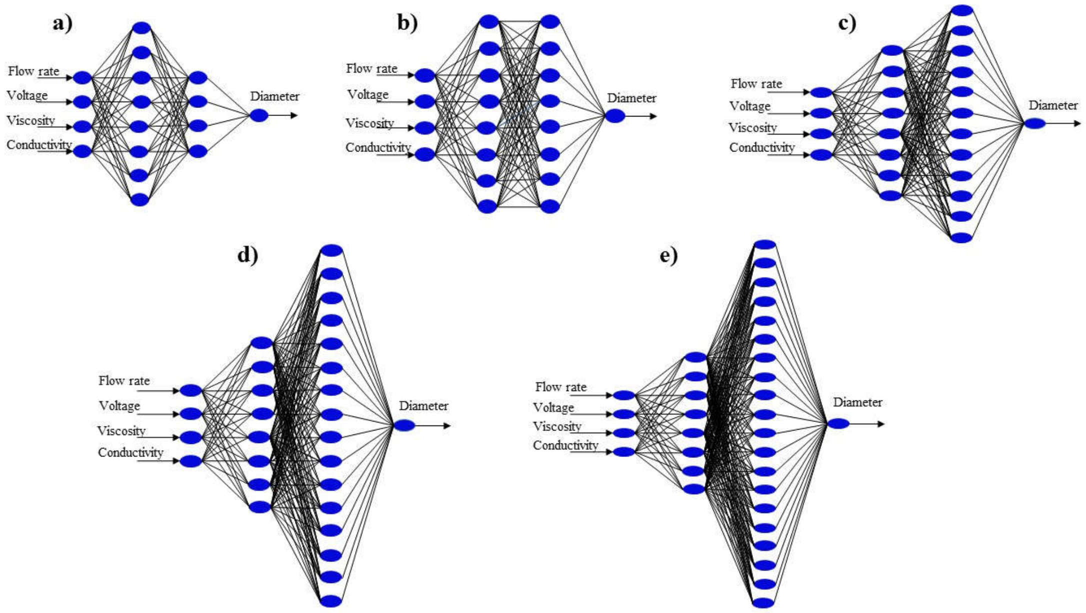

Figure S2. Configurations with two hidden layers.

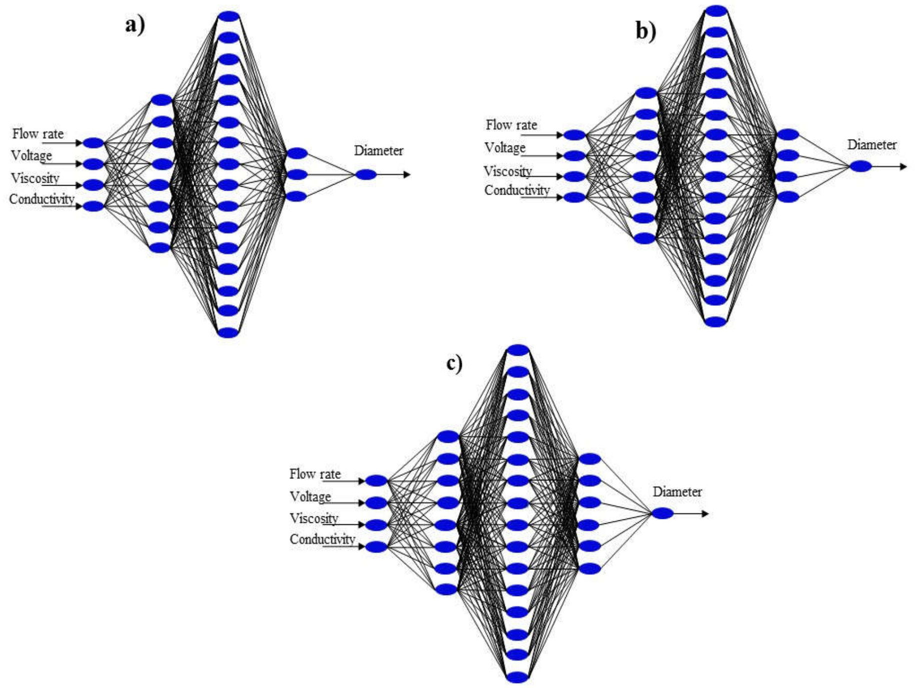

Figure S3. Configurations with three hidden layers.

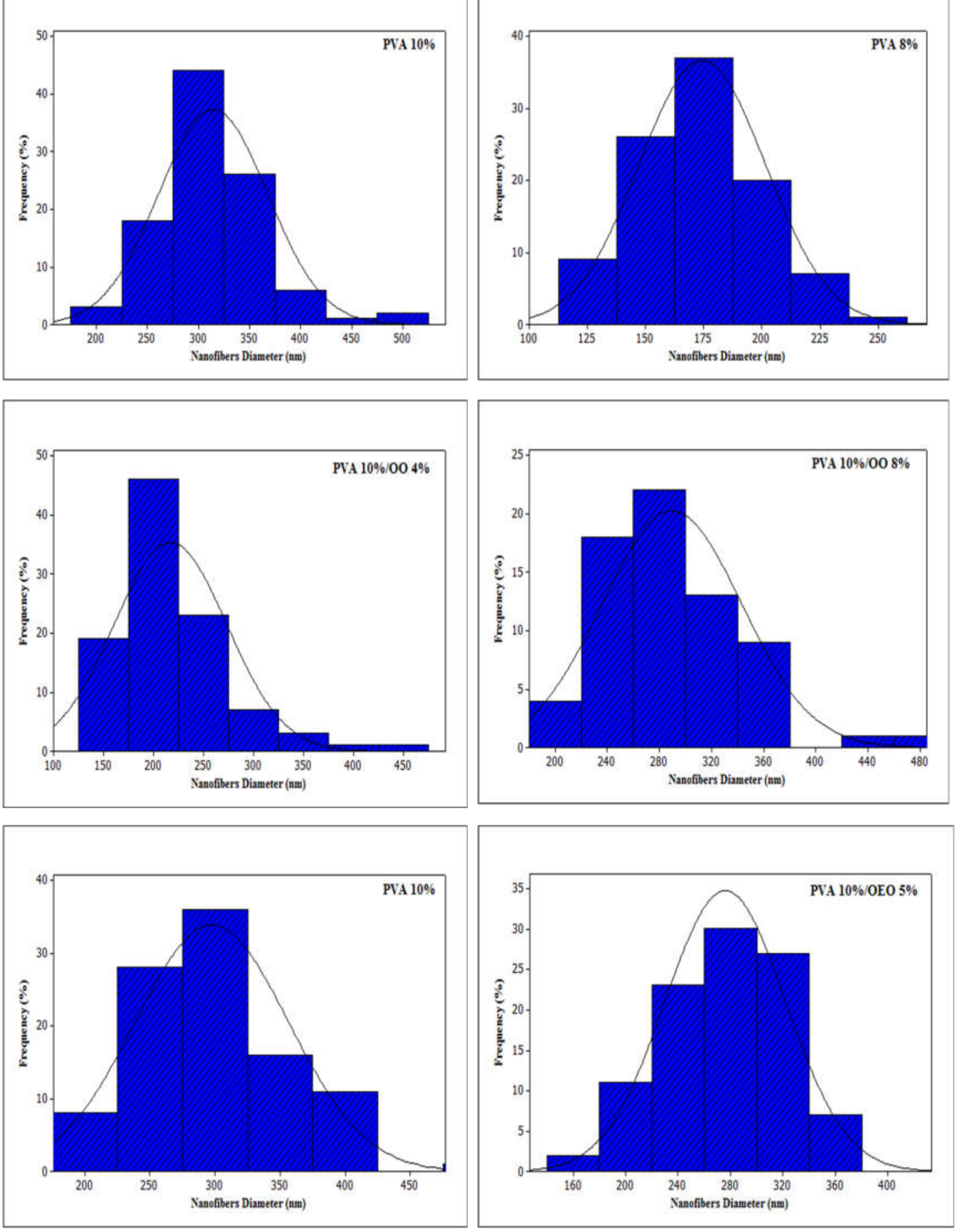
PVA 10%
PVA 8%
PVA 10%/OO 4%
PVA 10%/OO 8%
PVA 10%
PVA 10%/OEO 5%
Frequency (%)
Nanofibers Diameter (nm)

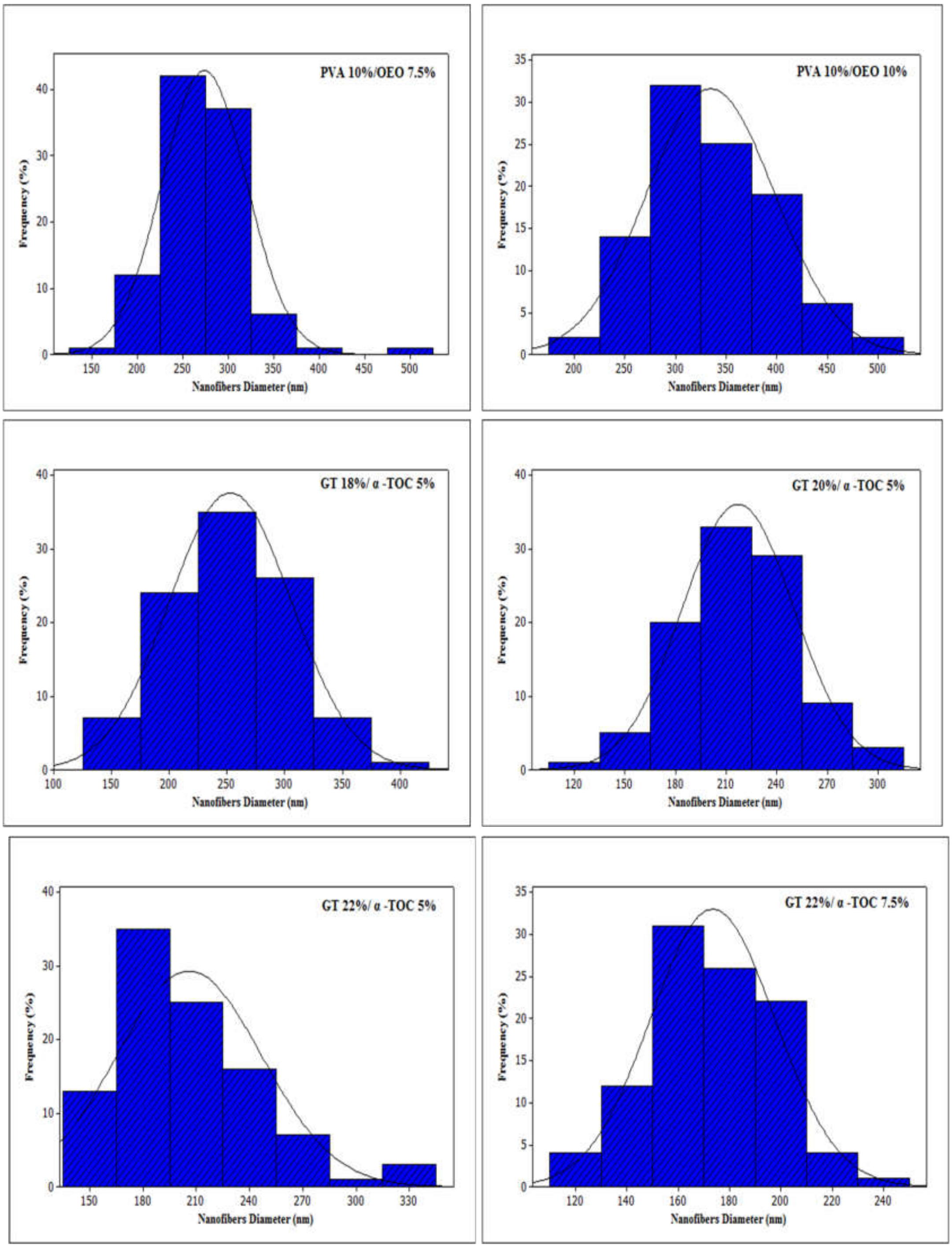
PVA 10%/OEO 7.5%
PVA 10%/OEO 10%
GT 18%/ α -TOC 5%
GT 20%/ α -TOC 5%
GT 22%/ α -TOC 5%
GT 22%/ α -TOC 7.5%
Frequency (%)
Nanofibers Diameter (nm)

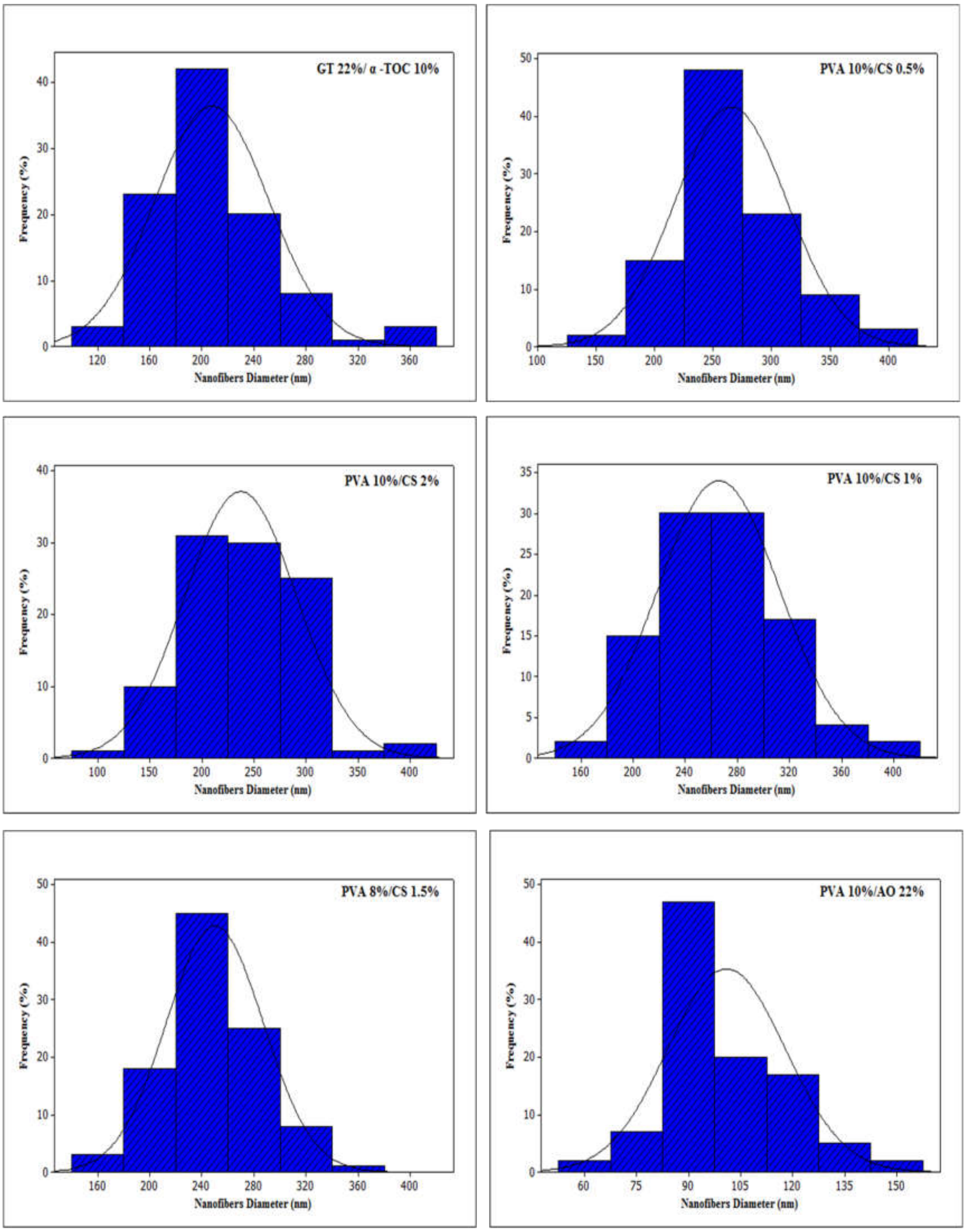
GT 22%/ α -TOC 10%
PVA 10%/CS 0.5%
PVA 10%/CS 2%
PVA 10%/CS 1%
PVA 8%/CS 1.5%
PVA 10%/AO 22%
Frequency (%)
Nanofibers Diameter (nm)

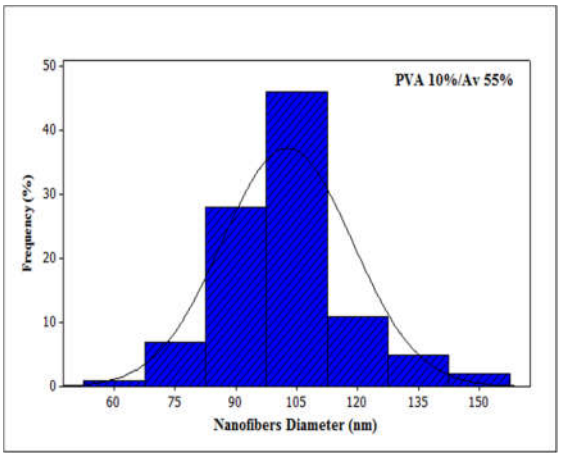

Figure S4. Diameter distribution of the electrospun nanofibers.
